# Harnessing electronic health records to study emerging environmental disasters: a proof of concept with perfluoroalkyl substances (PFAS)

**DOI:** 10.1038/s41746-021-00494-5

**Published:** 2021-08-11

**Authors:** Mary Regina Boland, Lena M. Davidson, Silvia P. Canelón, Jessica Meeker, Trevor Penning, John H. Holmes, Jason H. Moore

**Affiliations:** 1 Department of Biostatistics, Epidemiology and Informatics, Perelman School of Medicine, University of Pennsylvania, Philadelphia, USA; 2 Institute for Biomedical Informatics, University of Pennsylvania, Philadelphia, USA; 3 Center for Excellence in Environmental Toxicology, University of Pennsylvania, Philadelphia, USA; 4 Department of Biomedical and Health Informatics, Children’s Hospital of Philadelphia, Philadelphia, USA

**Keywords:** Risk factors, Epidemiology

## Abstract

Environmental disasters are anthropogenic catastrophic events that affect health. Famous disasters include the Seveso disaster and the Fukushima-Daiichi nuclear meltdown, which had disastrous health consequences. Traditional methods for studying environmental disasters are costly and time-intensive. We propose the use of electronic health records (EHR) and informatics methods to study the health effects of emergent environmental disasters in a cost-effective manner. An emergent environmental disaster is exposure to perfluoroalkyl substances (PFAS) in the Philadelphia area. Penn Medicine (PennMed) comprises multiple hospitals and facilities within the Philadelphia Metropolitan area, including over three thousand PFAS-exposed women living in one of the highest PFAS exposure areas nationwide. We developed a high-throughput method that utilizes only EHR data to evaluate the disease risk in this heavily exposed population. We replicated all five disease/conditions implicated by PFAS exposure, including hypercholesterolemia, thyroid disease, proteinuria, kidney disease and colitis, either directly or via closely related diagnoses. Using EHRs coupled with informatics enables the health impacts of environmental disasters to be more easily studied in large cohorts versus traditional methods that rely on interviews and expensive serum-based testing. By reducing cost and increasing the diversity of individuals included in studies, we can overcome many of the hurdles faced by previous studies, including a lack of racial and ethnic diversity. This proof-of-concept study confirms that EHRs can be used to study human health and disease impacts of environmental disasters and produces equivalent disease-exposure knowledge to prospective epidemiology studies while remaining cost-effective.

## Introduction

Environmental disasters are catastrophic events that occur and are the result of human activity (i.e., anthropogenic) and often inadvertent. These differ from natural disasters, which are presumed to be non-human caused (e.g., hurricane, volcano eruption), and intentional acts such as nuclear bombings. Environmental disasters often affect human health directly. On 10 July 1972 in Seveso, Italy, the Seveso Herbicide plant explosion occurred. This resulted in the highest known exposure to dioxin or 2,3,7,8-tetrachlorodibenzo-p-dioxin (TCDD) in residential populations^1–4^. Spontaneous abortions (i.e., miscarriages) increased 67.7% following the Seveso plant explosion, in addition fathers exposed tended to have more female offspring then expected^1–4^. More recently, the Fukushima-Daiichi nuclear disaster in Japan result in increased exposure to radiation and other chemicals. Review of medical and health records revealed no observed change in the fetal loss or anomaly rate following the Fukushima-Daiichi nuclear disaster^5^.

The typical epidemiology framework for studying environmental disasters includes conducting interviews of the exposed and affected populations, surveys to collect health outcomes, and in many cases review of health records. High-quality epidemiology studies use biomarkers of exposure, including serum concentrations of chemicals. However, these are expensive, typically costing over $100 per test and thereby limiting the sample size for study populations.

**Fig. 1:**
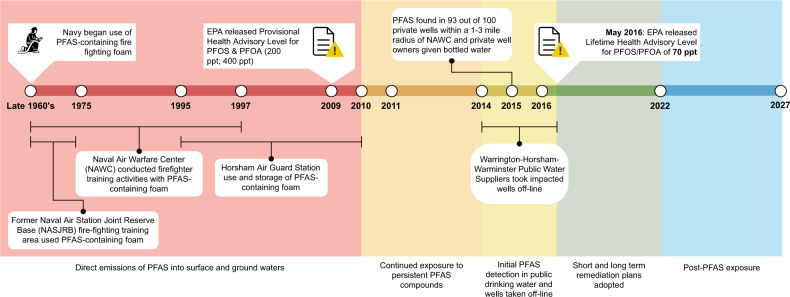
Horsham-Warminster-Warrington area PFAS exposure timeline. Two former military bases and an air guard station in this area had used PFAS-containing foam, aqueous film forming foam (AFFF): Willow Grove Former Naval Air Station Joint Reserve Base (NASJRB), Horsham Air Guard Station (HAGS) and Former Naval Air Warfare Center (NAWC). At these sites, AFFF with PFAS was used for a number of events, including firefighter training activities, fire suppression for aircraft crashes, and spills of the contaminants on site^11^. After initial discovery of PFAS in drinking water in 2014, contaminated wells were taken offline and public water service providers started purchasing supplemental water supply from the Forest Park Water Treatment facility through the North Wales Water Authority (NWWA). Bottled water or home water filtration systems were provided to those with contaminated private wells. May 2016, the EPA released a lower Lifetime Health Advisory Level of 70 ppt of combined PFOS/PFOA, leading to more closures of public and private water wells. Due to the half-life of PFAS species found in the community, post-PFAS exposure may be observed 2022–2027.

A recent environmental disaster in the United States of America (US) involves perfluoroalkyl substances (PFAS). PFAS anthropogenic persistent organic pollutants resistant to degradation and high heat and consist of different species of fluorinated organic compounds (**FOCs**). PFAS have surfactant properties and therefore are used in a variety of products, including firefighting foams, non-stick cookware, dental floss, fire and chemical resistant tubing, and more^6^. The two most common PFAS species found in serum in the US are perfluorooctanesulfonate (PFOS) and perfluorooctanoate (PFOA, or C8)^7–9^. The effect of PFAS on human health is only recently gaining awareness and therefore could be termed an emerging environmental disaster. PFAS were detected in the Philadelphia area, specifically in Horsham-Warminster-Warrington^10–12^ (Fig. 1). Overall, three of the top 10 water utilities with the highest nationally recorded PFAS levels were located close to Philadelphia: Horsham-Warminster-Warrington^12^.

As a result of research from C8 Science Panel in the Mid-Ohio communities, PFAS exposure is known to increase the risk for various health outcomes, including high cholesterol^13^, ulcerative colitis^14,15^, thyroid disease^13,14^, kidney cancer^16^, pregnancy-induced hypertension, and preeclampsia^17^. Several observational studies have found PFAS exposure associated with high serum uric acid levels^18–20^; epidemiologic evidence has linked elevated serum uric acid concentrations to hypertension^21^.

As mentioned previously, environmental epidemiologic studies typically rely heavily on interviews of the exposed and affected populations, surveys to collect health outcomes, recruitment of affected populations and in many cases manual review of health records. These studies require a painstaking amount of work and manual curation of specially designed datasets. For instance, the Pennsylvania Department of Health (DOH) conducted a recent PEATT Pilot Project study that was limited in sample size, selection and attrition rate: in the end only 204 participating adults, including 131 adult females total were included in the study^10^. Our study will be the largest so far in this community/population of exposed PFAS patients.

Data science has exploded these past few years with increasing numbers of studies seeking to incorporate ‘big data’ towards studying human health and disease. electronic health records (EHRs) contain millions of patient records across billions of visits, including details such as laboratory values, medications, procedures, diagnostic imaging, clinical notes, and demographic data. Therefore, EHRs contain a treasure-trove of data that could be useful for studying the health impacts of environmental disasters, such as PFAS exposure in a given population, if the appropriate informatics methods are applied.

Previously, several disease-environment correlation studies have been performed using EHRs. The first was Patel et al. who coined the term EWAS: Environment-Wide Association Study^22^. Others have studied environmental challenge in disease progression^23^, thyroid cancer hotspots in a rural community in Vermont^24^, and air pollution data in Italy^25^. We have also conducted several studies investigating the relationship between birth season (as a proxy for seasonal variance in environmental exposures at birth) and disease risk^26^. We conducted our initial study using New York City (NYC) data with novel findings that were validated in a separate EHR system^27^. We expanded our study to include six sites including South Korea and Taiwan^28^. This enabled us to develop a method that correlated birth season and trimester information with climate and pollution variables using location information^28^. We have also used our methods to study the effects of social exposures that can manifest as birth month relationships for attention deficit hyperactivity disorder (ADHD)^29^ and increased the number of datasets included in our assessment of female fertility and birth season to assess deeper and more complex multi-factorial environmental exposures^30^. We have found that prenatal exposure to fine air particulates increased the risk of atrial fibrillation later in life in humans^28^; this finding was supported by a study in a canine population^31,32^.

These methods demonstrate that informatics methods can be developed to overcome EHR biases^33,34^ and can be used to probe disease-environment interactions. However, they fall short in that they assess environmental exposures that are always present (e.g., air pollution) rather than the effect of an environmental disaster on human health and disease. An environmental disaster is more complex in that there is an exposure period, a remediation period and a post-exposure period (Fig. 1). Because the source of the PFAS exposure was from military property, the military has taken responsibility for the contamination, albeit the division of responsibilities between military branches complicated remediation^35^. Initially, contaminated wells were shut down, public water suppliers increased water purchasing, and owners of private contaminated wells were provided with in-home filtration or bottled water. State, Federal and Military agencies have made short and long term plans to remediate the area^36–38^.

This purpose of this study is to present a proof-of-concept detailing how EHRs can be used to study the impact of environmental disasters, such as PFAS exposure. We will compare the results generated from our EHR-only study to those that resulted from carefully curated epidemiology and government-based studies to demonstrate that we can effectively capture the health impacts of environmental disasters using EHR data only. Importantly, the Pennsylvania Department of Health (DOH) held a small study including only 204 adults and 131 females total exposed to PFAS^10^. Our study will be the largest so far in this community/population of exposed PFAS patients.

## Results

### EHRs can capture patients exposed to environmental disasters such as PFAS

**Fig. 2:**
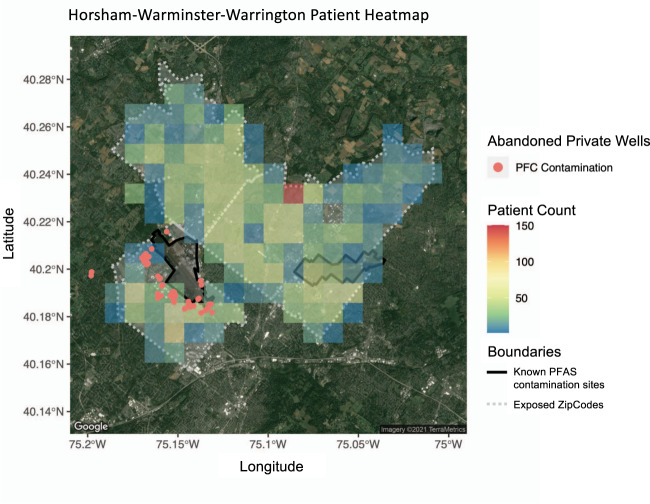
Heatmap detailing a subset of Penn Medicine patients living in Horsham-Warminster-Warrington, PA along with PFAS contamination sites and contaminated private wells. Select points are censored. The PFAS contaminated private wells are denoted with red points. The ZIP code boundaries are shown in gray-dashed bordering. Boundaries of the Former Naval Air Station Joint Reserve Base (NASJRB) and Former Naval Air Warfare Center (NAWC) are shown with black solid line bordering. The underlying base map is generated from the R package ggmap that uses a map from Maps Static API provided by Google Maps Platform. Additional layers and annotation provided by us.

**Fig. 3:**
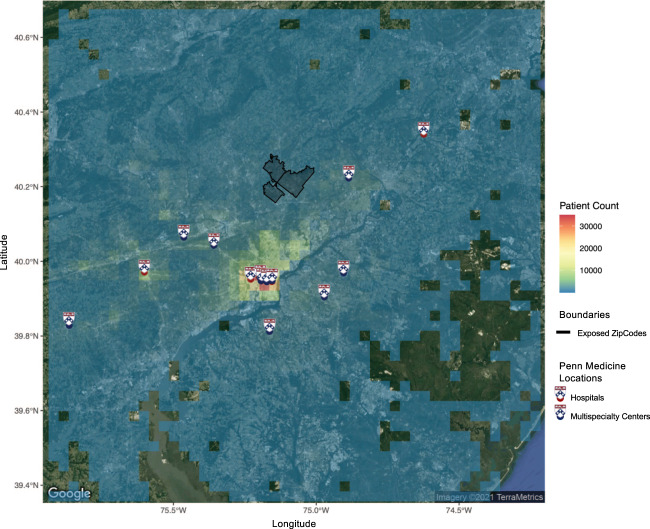
Heatmap detailing density of overall cohort of Penn Medicine patients living in the Greater Philadelphia Area. Select points are censored. The PFAS exposed ZIP code boundaries (Horsham-Warminster-Warrington, PA) are shown in black solid line bordering. Penn Medicine Locations are marked with The Penn Shield, red point for hospitals and blue point for multispecialty centers. This is not an exhaustive map of all Penn Medicine locations (i.e., rehabilitation facilities, and so forth), but represents the major hospitals and multi-specialty centers within PennMedicine as of July 2021. A total of 9 of the 19 Penn Medicine locations are within the city of Philadelphia. The underlying base map is generated from the R package ggmap that uses a map from Maps Static API provided by Google Maps Platform. Additional layers and annotation provided by us.

Using the zip code for individuals who were treated at Penn Medicine, we identified those living in Horsham-Warminster-Warrington. All of these women were included in our association analysis (results shown in subsequent section) with demographics given in Table 1. For mapping purposes, we linked address information recorded in the EHR to latitude and longitude. We identified the two major contamination sites in the Horsham-Warminster-Warrington area (depicted as black shapes in Fig. 2), along with the private well data denoting wells where PFAS contamination was reported for those exposed to the heavily polluted areas of Horsham-Warminster-Warrington (Fig. 2). Importantly, while we were unable to ascertain the socioeconomic status of individuals directly, we have some understanding of the socioeconomic status of those living in Horsham-Warminster-Warrington based on census information of those communities. For example, the median household income slightly above 80k and a poverty rate of 2.82%^39^. Therefore, this area is what could be called working class and neither extremely rich nor extremely poor. A map showing the larger PennMedicine catchment area is given in Fig. 3 along with logos to indicate the major PennMedicine hospitals and multispecialty centers.

**Table 1:** Demographics of our PFAS exposed vs. general inpatient population used in this study.

	PFAS exposed (*N* = 589)				General inpatient population (*N* = 149,882)	
Race	*N*		%		*N*	%
Asian	20		3.40%		5638	3.76%
Black or African American	23		3.90%		57,710	38.50%
Other^a^	21		3.57%		4315	2.88%
Unknown	19		3.23%		3702	2.47%
White	510		86.59%		78,692	52.50%
Ethnicity						
Hispanic	11		1.87%		7082	4.73%
Non-Hispanic	577		97.96%		141816	94.62%
Unknown	<5		<1%		1102	<1%
Age (Mean ±Standard deviation)^b^	56.28 ± 13.91				55.60 ± 18.72	

Neighborhood characteristics						
Income	Horsham	Warminster		Warrington		
Median household ($)	61,998	54,375		66,364		
Below poverty line (% of population)	2.4	5.3		2.6		
Reference	^39^	^61^		^62^		

Note 1: Racial and ethnic groups are displayed in alphabetical order.

Note 2: When a group contained <5 patients, it was reported as <5. Similarly, when percentages fall below 1%, we report as <1%.

^a^Other (Includes Other, Native Hawaiian or Other Pacific Islander, Mixed race, American Indian or Alaskan Native).

^b^Age is presented as average encounter age across all encounters.

### Validation of known disease-PFAS associations using clinical data from EHRs only

Women living the Horsham-Warminster-Warrington are exposed to PFAS at high levels. Therefore, we investigated whether or not we could replicate diseases known to be associated with high PFAS exposure among women visiting PennMed that lived in Horsham-Warminster-Warrington between 2010 and 2017 (during the highly exposed period). We investigated all diseases known to be elevated among women exposed to PFAS and compared the disease incidence to the background population. We found that high cholesterol; colitis and thyroid diseases (including multiple thyroid conditions) were elevated among women living in Horsham-Warminster-Warrington (Table 2). We used Steenland et al.’s comprehensive review^40^ to determine ranges of odds ratios (ORs) reported in literature for diseases linked with PFAS exposure. If multiple studies were found, we took the study with the lowest reported OR, which for high cholesterol was 1.38^19,40^. There are reports of cardiovascular disease and cerebrovascular disease increases among PFAS exposed but these studies were too small for general conclusions according to Steenland^40^.

**Table 2:** Association results for known PFAS exposed patients (Horsham-Warminster-Warrington) and diseases known to be associated with PFAS.

Related organ	Disease	Effect from Lit.	Ref.	Disease in EHR	Odds Ratio from EHR (95% CI; *P*-value)
Liver	High cholesterol	Odds ratio 1.38	^13,51^	Pure hypercholesterolemia	1.59 (CI = 1.093, 2.237; *p* = 0.013)
Kidney	Elevated uric acid	Odds ratio 1.00–1.47	^40^	Laboratory measure not in our dataset	
	Never assessed directly in literature			Proteinuria, unspecified	6.67 (CI = 1.351, 20.070; *p* = 0.011)
	Never assessed directly in literature			Complications of transplanted kidney	3.11 (CI = 1.130, 6.851; *p* = 0.015)
	Kidney cancer	Hazard ratio for increasing quartiles were 1.0, 1.23, 1.48 and 1.58	^16^		
Thyroid	Thyroid disease	Odds ratio 1.64–1.86		Malignant neoplasm of the thyroid gland	3.23 (CI = 1.651, 5.706; *p* < 0.001)
				Primary hyperparathyroidism	3.93 (CI = 1.061, 10.250; *p* = 0.021)
				Unspecified acquired hypothyroidism	1.53 (CI = 1.177, 1.967; *p* = 0.002)
	Ulcerative colitis	Adj. rate ratio 1.76–2.86	^15^	Toxic gastroenteritis and colitis	4.41 (CI = 1.188, 11.502; *p* = 0.014)
				Regional enteritis of unspecified site	3.11 (CI = 1.411, 5.985; *p* = 0.003)

With regards to renal or kidney function, we found the proteinuria was elevated. Elevated proteinuria is an independent risk factor for end-stage renal disease^41–43^. We did not find kidney cancer to be elevated among these women, but found that complications related to kidney transplants were elevated, which could be indicative of impaired renal function. Steenland et al.’s review^40^ found no relation between kidney function and PFAS exposure; however, another study^16^ reported a hazard ratio of 1.10 for kidney cancer for every 1 unit increase in PFOA in serum. Therefore, we looked for any kidney-related diagnoses that were nominally associated with PFAS exposure and found complications of transplanted kidney, which could indicate some impairment of kidney function among heavily exposed. Therefore, our results point to some potential relationship between renal function and PFAS exposure, however not the previously reported kidney cancer^16^. Importantly, Steenland et al.’s review^40^ found no relation between kidney function and PFAS exposure, suggesting that the relationship between kidney function and PFAS exposure is complex and requires further study.

Overall, these results demonstrate that clinical outcomes known to be due to high PFAS exposure in the literature are extractable from EHRs. In addition, it demonstrates that EHRs can be used to study the impact of environmental disasters on human health and disease without the need for specialized study recruitment and surveys.

## Discussion

Environmental disasters are large-scale anthropogenic events often with a fixed date—which for detection of PFAS in the Horsham-Warminster-Warrington area reportedly ranges from 29 October 2014 to 19 May 2016, with ~50 years of community exposure (Fig. 1). We demonstrate that EHRs can be used to study health outcomes following exposure to environmental disasters. Below, we discuss the many strengths of using EHRs to study environmental disasters and pollution in general over that of traditional epidemiology studies that require prospective patient recruitment.

Epidemiology studies focused on environmental disasters—such as PFAS exposure in Horsham-Warminster-Warrington are expensive and often limited to a particular disease area, such as cancer. Government agencies, such as the EPA and the Agency for Toxic Substances and Disease Registry (ATSDR), often need to perform public health assessments on hazardous sites facing designation as a Superfund or Brownfield site. However, these public health agencies face many resource and financial constraints when deciding what outcomes to study, the wash out period following exposure, and in which locations^44^. This leads to intense frustration in communities if their locations are not selected for further study, or if the outcomes they are most interested in are not explored—especially if they are already noticing alarming signs of deteriorating health among their loved ones. Even when a community is selected for study, it can be incredibly difficult for community members to wait for the results of the assessment, let alone the long process during which EPA and ATSDR decide on removal and remediation efforts. We should point out that the CDC did not select the Horsham-Warminster-Warrington site for future study likely due to budgetary constraints. In this current funding environment, studies using less expensive methods by harnessing EHR data become imperative.

The power of using EHRs to study environmental disasters such as PFAS, is that a wide range of health outcomes can be relatively quickly explored in an EWAS-style manner^22^. The purpose of this current study is to serve as a proof-of-concept to demonstrate that we can replicate the known diseases associated with PFAS exposure in gold standard epidemiologic studies. This is a first step towards further detailed high-throughput analyses on diseases that individuals are at increased risk for following PFAS exposure. Furthermore, we demonstrate that EHRs are a powerful tool that can be used by public health investigators who are interested in studying disparate health outcomes.

Socioeconomic analyses in the study of PFAS exposure and its subsequent effects on human health and disease are also important^45^. In this proof-of-concept, we ignore the socioeconomic factors because our desire was to replicate prior research on the health risks associated with PFAS exposure and the prior research often ignores socioeconomic co-contributors. A meta-analysis found that with a doubling of income the exposure to PFAS increased on average 10–14%^46^. This is contrary to the typical environmental justice hypothesis that higher income individuals have lower exposure to pollutants in general^46^. Many theories exist to explain this counter-intuitive result; some conclude that it could be due to routes of exposures (e.g., dental floss) or perhaps a result of the way many prior studies were conducted.

However, traditional epidemiology studies often require patient recruitment and consent and therefore are likely to result in inequities in part because recruiting representative populations is challenging^47^. Recruiting minority populations is especially challenging due to historical inequities and distrust^47^. Using EHRs to study environmental disasters, such as PFAS exposure, would address many of these issues. With a ready availability of clinical data from the EHR, a representative sample of the exposed population (in terms of race/ethnicity/sex/gender/various income levels) can be derived. This can mitigate some of the data collection and case ascertainment challenges experienced by traditional epidemiology studies.

Analyzing EHR data can enable elucidation of issues with specific datasets, for example if a clinical dataset is derived from one type of clinic (e.g., ‘oncology’) the quality of the health assessment for other diseases that are non-oncology related will be affected^48^. These dataset stratification issues could result in a biased assessment of certain exposure-disease related findings. Much work has been done to assess the bias of EHR methods and their portability from one clinic to the next^28,49,50^. However, many environmental studies consist of meta-analyses of results across different sites without exploring site-specific biases that may affect the results. These types of biases can be addressed using informatics methods applied to EHRs that contain rich phenotypic data that is not often available from epidemiology studies. This is another strength of using EHRs and informatics methods to study environmental disasters. In addition, we open a new area of research for informatics through the exploration of health effects following environmental disasters.

### Hypercholesterolemia

Our results for hypercholesterolemia are very similar to the literature with and OR of 1.38 being observed in the literature^13,51^ and 1.59 (*p* = 0013) being observed in our study. We did not have access to the exact laboratory values and therefore we should be underpowered to detect smaller non-diagnosis level changes in cholesterol levels. However, the PFAS exposure level is very high among our population and this likely explains our ability to detect these findings even using diagnosis code information alone. This demonstrates the strength of using EHRs for studying the health effects of emerging environmental disasters such as PFAS exposure in Horsham-Warminster-Warrington area.

### Kidney function

Proteinuria was shown to be an independent risk factor for end-stage renal disease^41–43^. We found that the diagnosis of proteinuria, unspecified was associated with PFAS exposure with an OR = 6.67. Of note, we do not have the laboratory value results in this analysis and therefore, we are relying on the diagnosis code for ‘proteinuria, unspecified’ (Table 2). We also found complications of transplanted kidney to be associated with PFAS exposure, which could indicate some impairment of kidney function among heavily exposed. Overall, our results point to some potential relationship between renal function and PFAS exposure, however not the previously reported kidney cancer^16^. Importantly, Steenland et al.’s review^40^ found no relation between kidney function and PFAS exposure, suggesting that the relationship between kidney function and PFAS exposure is complex and requires further study.

### Thyroid disease

Previous studies have found links between various thyroid hormonal levels and PFAS exposure. However, because we do not have access to thyroid hormone laboratory results, we used the presence/absence of various thyroid conditions. In the literature ORs were observed for women for thyroid disease (OR = 1.64) and taking medications related to thyroid disease (OR = 1.86) with slightly lower ORs for males^40^. We found that malignant neoplasm of the thyroid gland was elevated among women with OR = 3.23 (*p* < 0.001) and primary hyperparathyroidism was also elevated with an OR = 3.93 (*p* = 0.021). We also found that unspecified acquired hypothyroidism was elevated with an OR = 1.53 (*p* = 0.002), which was closer in risk size to those reported in previous studies^40^. Initially, it may seem conflicting that both hypothyroidism and hyperthyroidism condition codes demonstrated increased risk in our PFAS exposure sample. However, many individuals who are being treated for thyroid disease have either hypo- or hyper- thyroidism at various stages of their therapy while the medication levels are being titrated^52^. This could be the reason for our findings, and future work involves including laboratory values and medication histories for more detailed exploration in these findings. Importantly, the extremely high OR for malignant thyroid cancer in our population OR = 3.23 indicates the important difference between studying PFAS exposure as an occupation as in other studies versus our population, which was heavily exposed chronically over a long period of time.

### Kidney disease/cancer

A comprehensive review found no relation between kidney function and PFAS exposure^40^. However, due to another study^16^, we looked for any kidney-related diagnoses that were nominally associated with PFAS exposure and found complications of transplanted kidney (OR = 3.11), which could indicate some impairment of kidney function among heavily exposed.

### Colitis

A study found an adjusted rate ratio of 1.76–2.86 for ulcerative colitis among PFAS exposed individuals^15^. In our heavily exposed population, we found that toxic gastroenteritis and colitis had an OR = 4.41 (*p* = 0.014) and ‘regional enteritis of unspecified site’ had an OR = 3.11 (*p* = 0.003) indicated that gastrointestinal issues and colitis are at increased risk among heavily PFAS exposed individuals.

There are several limitations of our work. We investigated findings reported in the literature to determine if we could replicate these well-known findings. We successfully validated known PFAS-disease exposures. Inpatient EHR data often has bias towards patients who interact more with the healthcare system; these patients have more encounters and likely are sicker^53^. Our EHR data is sourced from inpatient clinic data and we compare our case population of PFAS exposed patients treated via the inpatient setting to other patients at PennMedicine from non-PFAS exposed areas also treated via the inpatient setting. Therefor e, our work should not have as strong of a bias towards sicker patients since we are comparing two similar populations. Other healthcare systems provide care for the Horsham-Warminster-Warrington community, which may lead to a demographic and geographic distribution bias in our cohort. Moreover, it is important to note that other environmental exposures can also cause many of these diseases. For example, Endocrine Disrupting Chemicals can disrupt the thyroid system (e.g., PCBs and Dioxins). At the former NAWC, wastes (e.g., paints, solvents, sludges from industrial wastewater treatment and waste oils) were generated over the years of use. In 1989, NAWC had been placed on the Superfunds program’s National Priorities List due to the threat to groundwater quality by eight disposal areas^54^; future work could consider human health effects associated with exposure to trichloroethylene and tetrachloroethylene. Our primary purpose was to determine if EHRs were useful in studying environmental disasters—given our ability to replicate the known PFAS-disease relationships, we conclude that they are a useful and cost-effective method of studying the health impacts of environmental disasters. Future work includes more sophisticated modeling to improve upon the state-of-the-art in terms of epidemiological and surveillance research. We aim to develop additional statistical methods that address for various sources of confounding in our populations to refine the state of the art in this field and also build new knowledge of PFAS exposure and its risks in terms of human health and disease.

From an informatics perspective, the alignment between diagnosis, symptom and condition codes found in the EHR and the diseases reported in these prior studies may not be exact. Future work includes our plan to link disease codes from ICD-9 and ICD-10 to higher-level disease categories using ontologies such as the Disease Ontology or the Human Phenotype Ontology to assist in linking these codes to exact disease entities (although many of these methods have other limitations). However, it is still likely that those entities may differ from what was reported in the literature by other researchers. Therefore, we also plan to expand our analysis to include laboratory values; however, this is challenging due to the many different laboratory-testing services that exist with different reference standards and the porting of information in the EHR usually occurs in PDF format. Therefore, this is the subject of future work. We also plan on using medication histories to determine treatment status (especially important for diseases/conditions like hypercholesterolemia and thyroid disease).

In addition, there is the limitation of address accuracy, we used patient zip code information—if this was incorrect then we may be misclassifying patients as cases. Also, we do not have information on whether or not patients were using bottled water or not— therefore, it is likely we are underestimating the true effect of the PFAS exposure on human health. In addition, we classified everyone living between 2010–2017 in the Horsham-Warminster-Warrington area as being exposed—this might not be the case if someone moved to the area in 2017 after the remediation period. This limitation also would lead us to underestimate the true effect of this exposure on human health.

Another limitation is that we are lacking socioeconomic status on the individual patients themselves. Importantly, while we were unable to ascertain the socioeconomic status of individuals directly, we have some understanding of the socioeconomic status of those living in Horsham-Warminster-Warrington based on census information of those communities. For example, the median household income slightly above $80,000 USD and a poverty rate of 2.82%^39^. Therefore, this area is what could be called working class and neither extremely rich nor extremely poor. Therefore, while we lack this information for our cohort, we do not expect it to drastically skew our results as our PFAS exposed patients are neither extremely poor nor extremely rich.

In conclusion, we were able to successfully validate many of the known findings related to PFAS exposure reported in the literature using our heavily exposed PFAS population located in Horsham-Warminster-Warrington. This demonstrates the utility for using EHRs for studying emerging environmental disasters such as PFAS exposure in Pennsylvania. The advantages of using EHRs are described including the greater access to diverse patient populations that may not typically sign up for a clinical trial or detailed occupational study. In addition, we are able to study patients for longer periods of time then are often available to researchers who enroll patients, which is another advantage of EHRs for this type of environmental research.

## Methods

### Overview of methods

This study involves the harmonization of data from the EPA, state and local water utilities and the Centers for Disease Control and Prevention (CDC) to appropriately capture the exposure—PFAS drinking water exposure. Once the exposure is properly categorized and assigned to a given latitude/longitude locations, we can correlate exposure with health outcomes using EHR data. We will describe these steps in detail below.

### Integrating diverse data sources to define PFAS ‘contaminated regions’

**Fig. 4:**
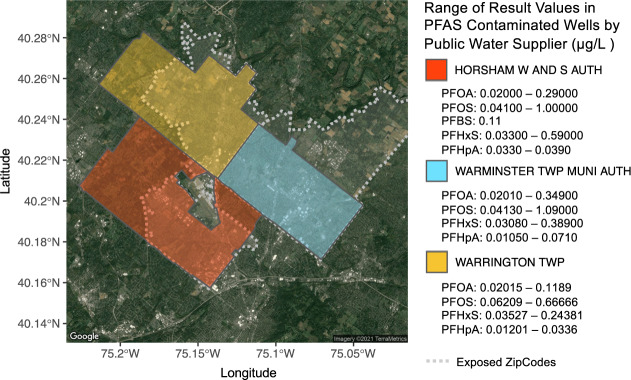
PFAS exposure levels as reported in public water utilities. Results over the minimum reporting level (MRL) defined by UCMR-3 are recorded in μg/L and null values indicate results less than the MRL. The MRL for each contaminant is as follows: PFOS (0.02), PFOA (0.04), PFBS (0.09), PFNA (0.02), PFHxS (0.03), and PFHpA (0.01). In all three public water systems, PFOS, PFOA, PFHxS and PFHpA were detected over the MRL. PFBS was solely detected above MRL in Horsham Water and Sewer Authority, while PFNA was not detectable above MRL in any of the three systems. Corresponding ZIP code boundaries are shown with gray-dashed bordering. The underlying base map is generated from the R package ggmap that uses a map from Maps Static API provided by Google Maps Platform. Additional layers and annotation provided by us.

The PFAS exposure in Horsham-Warminster-Warrington, PA was via the water supply. However, these three towns are located outside of Philadelphia city proper and therefore, some homes are on city/town water line while others are on private water wells. Exposure via the public water utilities (city/town water) is shown in Fig. 4.

#### City/town water

Information and data on PFAS contamination of public drinking water supplies are available via the Third Unregulated Contaminant Monitoring Rule (UCMR-3) database maintained by the EPA^48^. UCMR-3 monitoring occurred from 2013–2015 and included 6 PFAS species: namely PFOA, PFOS, PFNA, perfluorobutanesulfonic acid (PFBS), perfluoroheptanoic acid (PFHpA), and perfluorohexane sulfonic acid (PFHxS) for assessment monitoring using EPA-approved analytical methods. While these 6 PFAS species do not represent all possible PFAS species (Table 3), they are the most common and EPA provides this information freely to the public. Public Water Systems have a unique identification code (PWSID) and may contain several facilities at which sampling occurred. Boundaries of current public water suppliers’ (PWS) service areas are available from the PA Department of Environmental Protection (PA-DEP) Open Data Portal, which provides access to public non-sensitive GIS data^55^. The boundaries are approximate.

**Table 3:** PFAS terminology and species names.

PFAS type	Acronym	Species full name	Molecular structure	Observed half-life in humans^a^	Blood serum detected community exposure^b^
Perfluorocarboxylic acids (PFCA)	PFHpA	perfluoroheptanoic acid	C_7_HF_13_O_2_	0.17 y^57^	Y (0.4%)
	PFOA or C8	perfluorooctanoic acid	C_8_HF_15_O_2_	1.7–2.7 y^57,58^	Y (98.7%)
	PFNA or C9	perfluorononanoic acid	C_9_HF_17_O_2_	1.7–3.2 y^63^	Y (78.1%)
	PFDA	Perfluorodecanoic acid	C_10_HF_19_O_2_	4.0–7.1 y^63^	Y (6.0%)
Perfluorosulfonic acids (PFSA)	PFBS	perfluorobutane sulfonic acid	C_4_F_9_SO_3_H	0.12 y^57^	N
	PFHxS	perfluorohexane sulfonic acid	C_6_HF_13_SO_3_H	2.86–5.3 y^57,58^	Y (99.0%)
	PFOS	perfluorooctane sulfonic acid	C_8_HF_17_SO_3_H	2.91–3.4 y^57,58^	Y (100%)
PFAS precursors	MeFOSAA	2-(N-Methyl-perfluorooctane sulfonamido) acetic acid	C_11_H_6_F_17_NO_4_S	3.5 y^64^	Y (3.8%)

^a^There is significant interindividual variability and slower excretion observed for men than women^65^.

^b^Source: PEATT Pilot Project^10^.

#### Well water

Over one million domestic water wells are distributed across Pennsylvania. The Pennsylvania Groundwater Information System (PaGWIS) contains information on well water and springs^56^. Data in PaGWIS are from completed water well reports collected since the mid-1960s and deposited in a database since the 1980s. In addition, 55,000 records contained in PaGWIS are field-located wells recorded in the US Geological Survey (USGS) database^56^. The PaGWIS contains information on well abandonment allowing us to identify locations of abandoned private wells. When a well is abandoned the reason is reported as either: (a) PFAS contamination, (b) poor water quality or (c) other reason. We strictly used wells abandoned due to PFAS contamination. Unfortunately, this dataset does not contain information on the PFAS species (Table 3) or other contaminants found in the well. Private well sampling conducted by the Navy and Air National Guard is not available, however the sampling results were published via meeting minutes and reports for the Horsham-Warminster-Warrington community^11,37^.

### Linking individual-level exposure to PFAS ‘contaminated regions’ with EHRs

For the purposes of this proof-of-concept study, we focused on women living in the three towns that were exposed to extremely high levels of PFAS. These towns are Horsham, Warminster and Warrington referred to as Horsham-Warminster-Warrington throughout this study. These towns receive water supply from three of the top 10 water utilities with the highest nationally recorded PFAS levels in the entire US^12^. Using address information contained in the EHRs, we linked patients to their precise geolocation within the Horsham-Warminster-Warrington area for visualization purposes. We used ZIP code information for the three towns, Horsham-Warminster-Warrington, to extract patients in exposed regions for our association analyses. Public water sampling may not reflect exposure at the tap due to several factors in public water distribution (such as blending from several wells) and therefore we used binary exposure. We then extracted all diagnosis information contained in International Classification of Diseases (ICD) coding schema versions 9 and 10 for data collected between 2010 and 2017. These data were obtained during routine clinical care and stored in EHRs. Short term plans (e.g., bottled water, taking contaminated wells off-line) began after the EPA set the Lifetime Health Advisory Level in May 2016 (Fig. 1). We have data through 2017 and therefore all data is either pre-remediation or during the beginning of the remediation period. The biological half-life for PFAS species (shown in Table 3) ranges from years depending on the compound^57,58^: 1.7–2.7 years for PFOA, 2.91–3.4 year for PFOS, and 2.86–5.3 years for PFHxS. Therefore, all patients with EHR data between 2010 and 2017 found in this heavily PFAS-exposed region would be expected to have elevated PFAS levels. Because this research constitutes retrospective analysis of existing EHR data without further contact with patients, we obtained a waiver of consent. The Institutional Review Board (IRB) at the University of Pennsylvania approved this study.

### Statistical analysis: determining if known PFAS exposure-disease associations are captured using EHR clinical data alone

We obtained inpatient health records for patients treated at the Penn Medicine health system between 2010 and 2017. Our cohort consisted of 149,882 patients treated via the inpatient health system. Of these 589 patients were considered PFAS exposed as they lived in one of the three affected zip codes. Table 1 contains the demographic information for these patients for the overall inpatient population and the PFAS exposed population. Patients in the PFAS exposed group were more likely to report White race and less likely to report Black or African-American race when compared to the general population of inpatients at PennMedicine. Otherwise the racial/ethnic distribution was similar. We do not have access to socioeconomic information directly.

We then performed Fisher’s exact test to determine the association between disease/condition/symptoms codes having at least 100 patients diagnosed at PennMed. We compared the disease/condition/symptoms frequencies between those heavily exposed to PFAS (i.e., living in the Horsham-Warminster-Warrington towns) versus the overall cohort of patients treated at PennMed. Our approach was not ‘hypothesis-free’ in that we focused on diseases that were reported in the literature to be related to PFAS exposure. This allowed us to confirm if EHRs are able to capture the known PFAS-disease relationships established in the epidemiology literature. These known PFAS-disease associations were established using carefully curated epidemiology studies and therefore they serve as a ‘gold-standard’. Associations have been established between high PFAS exposure and high cholesterol^13^, ulcerative colitis^15^, thyroid disease^59^ and kidney cancer^16^. Importantly, this research is mainly with PFOA and PFOS rather than all PFAS species found in the Horsham-Warminster-Warrington area^60^. We will determine if we can effectively replicate all known PFAS-disease associations reported in the literature—new causal associations are not being investigated. We are excluding associations that are related to pregnancy (preeclampsia) as this requires capture of the pregnancy timeline. In addition, we are excluding testicular and prostate cancer, as our population is restricted to women.

The University of Pennsylvania’s Institutional Review Board approved this study.

### Reporting summary

Further information on research design is available in the Nature Research Reporting Summary linked to this article.

## Supplementary information

Reporting Summary

## Data Availability

This paper uses Electronic Health Records data (EHR) obtained from the Penn Medicine health system. This data is not freely available or shareable due to patient privacy concerns. Therefore the ‘raw’ patient data is not shareable. However, we are sharing our results in this paper and our methods are described.

## Code availability

The code for this study relies heavily on Electronic Health Records data (EHR) obtained from the Penn Medicine health system, which is not publicly available (see Data Availability statement). Inquiries with regards to the code or customization to fit your particular dataset are available upon request.
