# Supplementary material for: Harnessing electronic health records to study emerging environmental disasters: a proof of concept with perfluoroalkyl substances (PFAS)
Source: NPJ Digit Med. 2021 Aug 11;4:122. doi: 10.1038/s41746-021-00494-5 (PMC8357930; doi:10.1038/s41746-021-00494-5)
Supplement: Supplementary file 1 — Reporting Summary [file 41746_2021_494_MOESM1_ESM.pdf]

nature portfolio

Corresponding author(s): Boland

Last updated by author(s): Jun 11, 2021

Reporting Summary

Nature Portfolio wishes to improve the reproducibility of the work that we publish. This form provides structure for consistency and transparency in reporting. For further information on Nature Portfolio policies, see our Editorial Policies and the Editorial Policy Checklist.

Statistics

For all statistical analyses, confirm that the following items are present in the figure legend, table legend, main text, or Methods section.

n/a	Confirmed	
☐	☒	The exact sample size (n) for each experimental group/condition, given as a discrete number and unit of measurement
☐	☒	A statement on whether measurements were taken from distinct samples or whether the same sample was measured repeatedly
☐	☒	The statistical test(s) used AND whether they are one- or two-sided<br>*Only common tests should be described solely by name; describe more complex techniques in the Methods section.*
☐	☒	A description of all covariates tested
☐	☒	A description of any assumptions or corrections, such as tests of normality and adjustment for multiple comparisons
☐	☒	A full description of the statistical parameters including central tendency (e.g. means) or other basic estimates (e.g. regression coefficient) AND variation (e.g. standard deviation) or associated estimates of uncertainty (e.g. confidence intervals)
☐	☒	For null hypothesis testing, the test statistic (e.g. F, t, r) with confidence intervals, effect sizes, degrees of freedom and P value noted<br>*Give P values as exact values whenever suitable.*
☒	☐	For Bayesian analysis, information on the choice of priors and Markov chain Monte Carlo settings
☒	☐	For hierarchical and complex designs, identification of the appropriate level for tests and full reporting of outcomes
☐	☒	Estimates of effect sizes (e.g. Cohen's d, Pearson's r), indicating how they were calculated

Our web collection on statistics for biologists contains articles on many of the points above.

Software and code

Policy information about availability of computer code

Data collection	These data are part of medical records from Electronic Health Records and therefore cannot be provided to readers directly. Patients received care at PennMedicine and their data were used in this retrospective study.
Data analysis	The Data Analysis was performed in EHR records and therefore this code is not readily available to all readers as much of the code involves using the records directly and therefore is not readily usable to others. In all possible places we used established R software packages (e.g., fisher exact test, and so forth) and these are available to others.

For manuscripts utilizing custom algorithms or software that are central to the research but not yet described in published literature, software must be made available to editors and reviewers. We strongly encourage code deposition in a community repository (e.g. GitHub). See the Nature Portfolio guidelines for submitting code & software for further information.

Data

Policy information about availability of data

All manuscripts must include a data availability statement. This statement should provide the following information, where applicable:

- Accession codes, unique identifiers, or web links for publicly available datasets
- A description of any restrictions on data availability
- For clinical datasets or third party data, please ensure that the statement adheres to our policy

Provide your data availability statement here.

Field-specific reporting

Please select the one below that is the best fit for your research. If you are not sure, read the appropriate sections before making your selection.

☒ Life sciences ☐ Behavioural & social sciences ☐ Ecological, evolutionary & environmental sciences

For a reference copy of the document with all sections, see nature.com/documents/nr-reporting-summary-flat.pdf

Life sciences study design

All studies must disclose on these points even when the disclosure is negative.

Sample size	Described in the paper - demographics given in Table 2.
Data exclusions	No data were excluded. All steps included in the paper.
Replication	This population we are studying is a heavily exposed PFAS population. Our replication is to compare our findings to the literature in terms of our known results - we were able to verify that we can use EHRs for this purpose as many known findings were replicated.
Randomization	This is a retrospective cohort study. Therefore no randomization was performed.
Blinding	This is a retrospective cohort study. Therefore no blinding was performed.

Reporting for specific materials, systems and methods

We require information from authors about some types of materials, experimental systems and methods used in many studies. Here, indicate whether each material, system or method listed is relevant to your study. If you are not sure if a list item applies to your research, read the appropriate section before selecting a response.

Materials & experimental systems

n/a	Involved in the study	
☒	☐	Antibodies
☒	☐	Eukaryotic cell lines
☒	☐	Palaeontology and archaeology
☒	☐	Animals and other organisms
☐	☒	Human research participants
☒	☐	Clinical data
☒	☐	Dual use research of concern

Methods

n/a	Involved in the study	
☒	☐	ChIP-seq
☒	☐	Flow cytometry
☒	☐	MRI-based neuroimaging

Human research participants

Policy information about studies involving human research participants

Population characteristics	We obtained existing clinical EHR records for females who received treatment at PennMedicine between 2010-2017 and binned them into exposed PFAS areas based on mailing address information. This is described in detail in the methods with demographics provided in Table 2.
Recruitment	This was a retrospective study on existing data and therefore a waiver of consent was obtained. We did not recruit patients to this trial explicitly.
Ethics oversight	IRB approval was obtained.

Note that full information on the approval of the study protocol must also be provided in the manuscript.
